# Grayken lessons: between a rock and a hard place? A 37-year-old man with acute liver injury while enrolled in a managed alcohol program for severe alcohol use disorder

**DOI:** 10.1186/s13722-023-00370-5

**Published:** 2023-03-06

**Authors:** Thomas D. Brothers, Alexander Y. Walley, Helen Rivers-Bowerman, Magnus McLeod, Leah Genge

**Affiliations:** 1 Department of Medicine, Dalhousie University, Halifax, NS Canada; 2 Institute of Epidemiology and Health Care, UCL Collaborative Centre for Inclusion Health, University College London, London, UK; 3 Grayken Center for Addiction, Clinical Addiction Research and Education (CARE) Unit, Section of General Internal Medicine, Department of Medicine, School of Medicine and Boston Medical Center, Boston University, Boston, MA USA; 4 Mobile Outreach Street Health (MOSH), North End Community Health Centre, Halifax, NS Canada; 5 Department of Family Medicine, Dalhousie University, Halifax, NS Canada

**Keywords:** Alcohol use disorder, Harm reduction, Substance-related disorders, Managed alcohol programs, Hepatitis, Substance use disorders, Addiction medicine

## Abstract

Managed alcohol programs aim to reduce health and social harms associated with severe alcohol use disorder. Here, we describe a young man with severe alcohol use disorder enrolled in a managed alcohol program, who was admitted to hospital with acute liver injury. Fearing that alcohol was contributing, the inpatient care team discontinued the managed alcohol dose in hospital. He was ultimately diagnosed with cephalexin-induced liver injury. After consideration of risks, benefits, and alternative options, the patient and care team jointly decided to restart managed alcohol after hospital discharge. With this case, we describe managed alcohol programs and summarize the emerging evidence-base, including eligibility criteria and outcome measures; we explore clinical and ethical dilemmas in caring for patients with liver disease within managed alcohol programs; and we emphasize principles of harm reduction and patient-centered care when establishing treatment plans for patients with severe alcohol use disorder and unstable housing.

## Patient information

Mr. S, a 37-year-old man, was admitted to hospital with acute liver injury.

Mr. S began drinking alcohol more than 20 years earlier and identified as “an alcoholic since I was a teenager”. Three years before this admission, Mr. S had a period of abstinence from alcohol while incarcerated and then resumed drinking upon release. In a typical day, he drank 1.18L (40 oz) of 40% alcohol by volume (ABV) liquor (equivalent to 27.8 Canadian standard drinks; around CAD$40.00 [USD$30.65]), when he could afford it. Otherwise, he would drink two 750 mL (25 oz) bottles of 20% ABV fortified wine (17.6 standard drinks; CAD$22.00 [USD$16.86]) and one 1000 mL (33.8 oz) bottle of 26% ABV alcohol-based mouthwash (15.3 standard drinks; CAD$9.00 [USD$6.90]), equivalent to total 32.9 standard drinks) daily.

Mr. S experienced worsening health and social harms related to alcohol use, consistent with severe alcohol use disorder. He was frequently arrested by police for public intoxication. In the year before this hospital admission, Mr. S had seven emergency department (ED) presentations related to alcohol intoxication, withdrawal, or alcohol-associated injuries. He reported withdrawal-related seizures every 2–4 weeks over the prior year, with the most recent occurring 2 months before this admission. During this time, he experienced unstable housing, staying in rooming houses, congregate shelters, and sleeping outdoors.

Three months before this hospital admission, Mr. S told his primary care physician at a local community health centre that he wished to decrease his alcohol intake. His physician referred him to inpatient withdrawal management [1, 2], where he was admitted after 2 weeks on a waiting list. The following day he left before medically advised, but with a plan to continue outpatient counselling. He resumed drinking his typical amount.

One month before this hospital admission, Mr. S accessed permanent, supportive housing through a Housing First program and enrolled in a managed alcohol program (MAP) affiliated with his community health centre [3–5]. He was dispensed twelve 12 oz cans of 6% ABV strong beer per day (14.4 Canadian standard drinks) to his apartment. He was also provided access to a case manager, outreach nurses, and a social worker who formed a multidisciplinary team along with his primary care physician. During this first month in MAP, Mr. S maintained his housing, had no police involvement, and had no ED visits related to alcohol. He also received a course of cephalexin for cellulitis in his arm.

On the day of this hospital admission, his case manager and outreach nurse noticed jaundice. Mr. S described feeling fatigue, nausea, and abdominal pain for several days with decreased food and fluid intake. They brought him to the hospital.

At the hospital, his liver enzymes and bilirubin were elevated with a normal international normalized ratio (INR; see Table 1). Fearing alcohol-associated hepatitis, the inpatient team discontinued Mr. S’ MAP regimen. Although alcohol was on the hospital formulary, the hospital did not have a protocol for continuing MAP among admitted patients.

**Table 1:** Laboratory and other diagnostic data for a 37-year-old man with severe alcohol use disorder admitted to the hospital for acute liver injury

Liver enzymes and function tests (normal range)	During 5 years before hospital admission^a^	Hospital Day 1	Hospital Day 2	Hospital Day 15
ALT (< 54 U/L)	45–256	171	141	91
AST (5–45 U/L)	54–126	836	631	162
ALP (38–150 U/L)	61	394	357	140
GGT (< 49 U/L)	27–93	3532	3008	845
Total Bilirubin (< 1 mg/dL)^b^	0.58	10.41	14.85	3.16
INR (0.8–1.2)	1.0	1.3	1.8	1.0
Other serology				
HCV antibody(+), viral load(−)				
Hemochromatosis: C282Y/H63D negative				
Liver biopsy				
Primarily cholestatic injury, not in keeping with primary alcoholic hepatitis				
Moderate steatosis, mild active steatohepatitis, heavy iron staining				
Stage 3 (out of 4) fibrosis				
MRCP				
No evidence of biliary obstruction				
Hepatic steatosis, smooth liver contour, no cirrhosis				

*ALT* Alanine transaminase, *U/L* units per litre, *AST* Aspartate transaminase, *ALP* Alkaline phosphatase, *GGT* gamma-glutamyl transferase, *INR* International Normalized Ratio, *MRCP* Magnetic resonance cholangiopancreatography

^a^Range of values during 5 years, or single value if only one available

^b^to convert bilirubin to umol/L, multiply these values by 17.1

The admitting team noted Mr. S to be confused and inattentive, and considered a differential diagnosis including alcohol-withdrawal delirium, delirium from medical causes like infection, hepatic encephalopathy, and Wernicke encephalopathy [6]. He was given thiamine, folic acid, and a multivitamin, and his alcohol withdrawal symptoms were managed with lorazepam following a symptom-triggered dosing protocol based on the Clinical Institute Withdrawal Assessment for Alcohol, revised (CIWA-Ar) [7].

Mr. S’ medical history was otherwise significant for opioid use disorder (in sustained remission, on methadone opioid agonist therapy), cocaine use disorder (in early remission, last smoking cocaine 3 months prior), tobacco use disorder (active), attention deficit hyperactivity disorder (treated with methylphenidate) and a history of hepatitis C virus infection (spontaneously cleared). He took no other regular medications. His bloodwork showed persistent, mild elevations in liver enzymes over the several preceding years (see Table 1), raising a question of chronic liver disease. He received social income assistance and lived with a supportive partner who did not drink alcohol.

The consulting hepatologist noted that Mr. S’ degree of AST elevation (in this case, 18–25 times the upper limit of normal) and ALP elevation were not typical of alcohol-associated hepatitis, and arranged a liver biopsy and magnetic resonance cholangiopancreatography (MRCP) to clarify the underlying etiology of the acute liver injury.

**Fig. 1:**
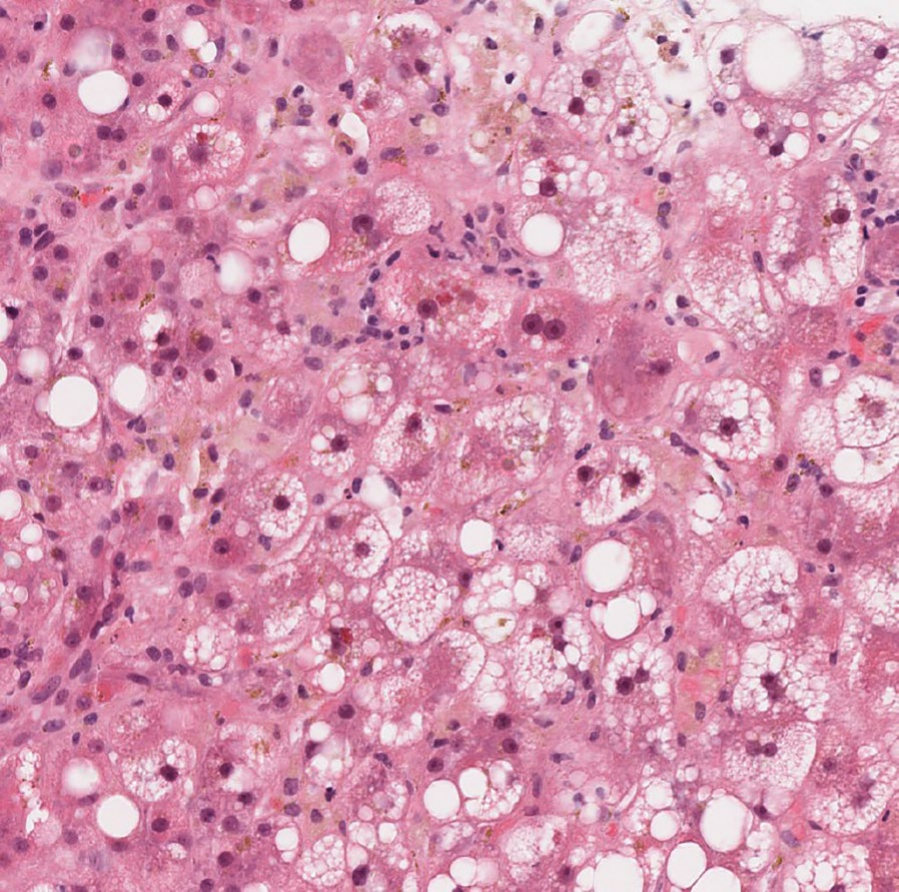
Histopathological examination of nontargeted liver biopsy of a 37-year-old man with severe alcohol use disorder admitted to the hospital for acute liver injury. The image shows lobular cholestasis (mostly hepatocellular) with feathery degeneration and bile-laden macrophages

The liver biopsy demonstrated a cholestatic injury pattern, which was not suggestive of alcohol-associated hepatitis (see Fig. 1). The biopsy and MRCP showed stage 3 fibrosis in keeping with chronic, non-cirrhotic, alcohol-associated liver disease. Considering the history and other causes of liver injury, the hepatologist believed this cholestatic liver injury was drug-induced from cephalexin. Cephalosporin-associated liver injuries are rare and typically recover spontaneously within 4–8 weeks [8]. Mr. S was advised to avoid cephalexin in the future and try to avoid alcohol as long as possible (at least until his bilirubin normalized).

Over 10 days in hospital, Mr. S’ delirium resolved and his liver enzymes and bilirubin improved (see Table 1). The admitting team made a referral to the hospital’s addiction medicine consultation service [9, 10] to assist with discharge planning and management decisions about whether to restart MAP or pursue an alternative care strategy for alcohol use disorder. The addiction medicine consultant assessed Mr. S and obtained collateral history from his partner, MAP case manager, and MAP nurse.

When asked what he thought would be helpful, Mr. S said: *“I would like to stay in MAP, I think it is helping me… I don’t think I could go without alcohol.”* His stated goals were to stay in housing, to avoid non-beverage alcohol [11–13], and work towards long-term physical and mental health. Mr. S and his partner both emphasized that he had tried abstinence from alcohol several times in the past 2 years, and each of these resulted in relapse, binge use, and physical harm. Mr. S did not believe that his recent completion of alcohol withdrawal management in hospital would meaningfully improve his odds of success with an abstinence-based approach. He understood and appreciated that if he continued to drink alcohol, he would likely soon develop cirrhosis.

Mr. S, his partner, the MAP team, his primary care physician, and the addiction medicine physician all supported reinitiating MAP. On the day of hospital discharge, the MAP team met Mr. S his apartment and provided him with eight 12 oz cans of 6% ABV strong beer per day (9.6 Canadian standard drinks). There was a plan to titrate back up to his previous dose of 12 cans (14.4 standard drinks), if he experienced ongoing withdrawal symptoms or cravings for alcohol.

Five months after hospital discharge (and 6 months after starting MAP), the patient remained enrolled in MAP and maintained his housing. He had one further ED visit for abdominal pain. This represents two total ED visits in 6 months on MAP, compared to seven in the year before enrolling in MAP (around 3.5 visits per 6 months). He had no further police contacts and no alcohol-related ED visits. An outpatient liver ultrasound showed moderate steatosis with mildly nodular contour and no ascites. His liver enzymes showed continued improvement (AST 174, down from a peak of 700; ALT 42; alkaline phosphatase 102; bilirubin 1.18 mg/dL).

Mr. S later told the MAP team that he had been drinking alcohol in hospital but was afraid to tell the inpatient care team because he believed it was not allowed and would affect his care.

## Key case questions

### 1. What are managed alcohol programs?

Managed alcohol programs (MAPs) are a harm reduction practice, aiming to reduce health and social harms associated with severe alcohol use disorder by providing a consistent supply of beverage-grade alcohol [3, 4, 14]. They are often offered alongside housing programs, with other social and health supports [3]. MAPs were initially developed in response to a crisis of outdoor freezing deaths among people deprived of housing, including people who were denied shelter because they were intoxicated [3]. Eligibility criteria vary by program. MAPs typically enroll people with severe alcohol use disorder who experience significant harms from binge drinking (e.g. severe intoxication, alcohol poisoning, injury, freezing, or assault) [3, 15, 16]. This includes people deprived of housing and people who drink non-beverage alcohol (e.g. mouth wash, hand sanitizer, or vanilla extract) that are low cost with high alcohol content [11–13, 17]. MAPs aim to provide an inclusive alternative to abstinence-only services and housing for people with severe alcohol use disorder [4, 11].

MAPs dispense beverage-grade alcohol at regular intervals, ranging from every hour (with witnessed consumption) to once daily (with unwitnessed consumption spread throughout the day). Dosing strategies for alcohol involve shared decision-making and typically aim to maintain or reduce overall alcohol intake; prevent alcohol withdrawal; reduce or eliminate non-beverage alcohol consumption; and facilitate access to health and social supports. Programs often have protocols to prevent severe intoxication and policies to discourage drinking alcohol outside of the program. Models of care vary, but typically offer connections to health care, case managers, and/or social workers [3]. Residential MAPs (where managed alcohol is paired with housing) may be at a “single site”, “scattered site” between multiple locations, or offered within congregate shelters. There are also non-residential MAPs. Some MAPs are clinician-led within a “medical model”, while others are community-led in a non-medical model [12]. Several Canadian hospitals have policies to support MAP, to help facilitate inpatient care (though Mr. S’ hospital, in this case, did not) [18, 19].

The Canadian Managed Alcohol Program Study (CMAPS) recognizes 39 MAPs across Canada [20]. MAPs have expanded during the COVID-19 pandemic, including temporary, emergency MAPs to facilitate physical distancing in Halifax, Nova Scotia, Canada [5]; San Francisco, California [21–23] and Juneau, Alaska, USA [24]; and Sydney, New South Wales, Australia [25]. To our knowledge, the programs in San Francisco and Juneau are the only MAPs in the United States. We are not aware of policy or regulatory restrictions preventing the expansion of MAPs in countries where alcohol is legal and regulated [26–28]. Limited uptake in the United States to date may be due to other factors like cost and differing attitudes towards addiction and harm reduction [26, 27, 29, 30]. Operational guidance for implementation of managed alcohol programs is provided in a recent publication from the Canadian Institute for Substance Use Research and the British Columbia Centre on Substance Use [15].

### 2. Are managed alcohol programs safe and effective?

Several studies identify potential improvements in health and social outcomes with MAPs, and evidence of no clear short-term harms [26]. Uncontrolled, before-and-after program evaluations suggest improvements in quality of life and maintaining housing, decreased use of non-beverage alcohol, reductions in ED and acute care hospital use, and reductions in police contacts [3, 11, 31–33]. One study found this resulted in cost savings for participants compared to time before they entered MAP, and also compared to a “treatment-as-usual” group [34]. There are no long-term studies comparing the health of people enrolled in MAP to similar people with hazardous drinking. A recent scoping review found no studies assessing the potential benefits or harms of MAP on liver disease, cancer, hypertension, or heart disease [26].

Stockwell and colleagues conducted a longitudinal, matched cohort study across six Canadian cities, comparing MAP participants to locally recruited controls who met MAP criteria but did not enrol [16]. Over 12 months, MAP and control participants reported consuming similar total amounts of alcohol per day, on average; however, MAP participants consumed fewer drinks per day spread out over more days per month. MAP participants reported fewer health and social harms compared to the control group within the first month and at 6 months follow-up, but both groups experienced similarly reduced harms by 12 months. This absence of a persistent difference may be explained by “regression-to-the-mean” for both MAP and control groups; i.e., people who are eligible for MAP might be experiencing atypically severe periods of alcohol-related harm that with time alone will improve towards less extreme levels. A post hoc exploratory analysis suggested most improvement among MAP participants was at programs with policies to limit outside drinking [16]. A retrospective cohort study using linked hospital records among a subset of this larger group found that MAP participants spent less time in hospital compared with locally recruited control participants who did not in enroll in MAP, but had similar rates of death and ED visits [35].

Pauly and colleagues conducted a qualitative case study to explore experiences of 53 MAP participants, four past participants, and 50 staff [4]. Prior to enrolling in MAPs, participants describe a pattern of “street survival”, cycling through multiple arenas (health, legal, housing/shelters) that required abstinence to receive help. MAPs can disrupt this cycle and provides safe environments for reconnection with family, healing/wellness, and other priorities [4].

MAPs incorporating culturally-relevant programming and culturally-safe practices may also be particularly helpful for Indigenous people, who face unique and disproportionate alcohol-related harms due to structural racism and settler-colonialism [36–38]. Several MAPs in Canada incorporate programming and activities provided by Indigenous organizations, including traditional forms of art, drumming, cooking traditional foods, feasts, use of sacred Indigenous medicines, smudging and prayer [3].

Overall, MAPs appear to be feasible, acceptable, and beneficial to some people with severe alcohol use disorder, especially in the context of unstable housing. Existing research provides promise that MAPs are associated with a safer pattern of alcohol consumption, with less binge drinking. A key benefit is that MAPs facilitate access to housing and services that are otherwise unavailable to people in settings where abstinence is required [3, 12]. Further research is needed on potential long-term health benefits and harms, and on understanding which MAP policies and practices are most beneficial to which participants (e.g., specific inclusion or exclusion criteria; voluntary policies to limit drinking outside the program) [16]. Further research is also needed to guide providers and participants on ongoing alcohol use outside of MAPs [39], especially in the setting of underlying liver disease. Understanding the direct effects of managed alcohol, beyond the benefits of supportive housing (provided alongside many MAPs) is still unclear.

### 3. What are clinical considerations of managed alcohol programs for people with liver disease?

Little evidence exists on the impact of MAPs on liver health, compared to people with severe alcohol use disorder who do not enroll [16, 40]. In their longitudinal study [16], Stockwell and colleagues describe trends in liver enzymes and bilirubin measurements for patients who enrolled in MAP (they did not obtain bloodwork from local controls). As summarized in Table 2, liver enzymes were generally stable after enrolling in MAP and worsened when people left the program, though changes were not statistically significant. Reasons for leaving MAP are not provided, and might have included binge drinking outside the program. The bilirubin values suggest that participants in this study were not representative of people with decompensated cirrhosis or alcohol-associated hepatitis. A case report by Hill and colleagues described a MAP participant with cirrhosis whose liver function further deteriorated when MAP was discontinued in hospital and the patient returned to binge, non-beverage alcohol use [40].

**Table 2:** Average liver enzymes and bilirubin among managed alcohol program (MAP) participants before entry to MAP, during MAP, and after discontinuing MAP, in the longitudinal study by Stockwell at el. [16]

Liver enzyme and function tests (normal range)	Before MAP, mean (95% CI)^a^	On MAP, mean (95% CI)^a^	Off MAP, mean (95% CI)^a^
ALT (7–56 U/L)	56 (38–74)	44 (22–67)	64 (47–82)
AST (5–40 U/L)	61 (40–82)	65 (39–91)	96 (76–117)
GGT (5–65 U/L)	221 (113–328)	266 (0–576)	492 (134–851)
Bilirubin (< 1 mg/dL)	0.64 (0.41–0.88)	0.82 (0.12–1.46)	1.11 (0.64–1.52)

*MAP* managed alcohol program, *CI* confidence intervals, *ALT* alanine transaminase, *U/L* units per litre, *AST* Aspartate transaminase, *GGT* gamma-glutamyl transferase

^a^Means and 95% confidence intervals generated from longitudinal mixed linear regression models

^b^to convert bilirubin to umol/L, multiply these values by 17.1

Stability or improvements in liver enzymes while on MAP may reflect reductions in non-beverage alcohol use and/or reductions in heavy binge drinking (with alcohol consumption spread out over more days per month). Further research is needed, but these data suggest liver disease should not be an absolute contraindication to MAP. MAP participants with chronic liver disease should be offered preventive care to reduce additional insults to the liver, including vaccination against hepatitis A and B virus infection (for those who are all not already immune). How to best monitor liver health among MAP participants is still unclear.

**Table 3:** Lessons learned from a case of a 37 year-old hospitalized for acute liver injury while enrolled in a managed alcohol program for severe alcohol disorder

1. Clinicians should be mindful of premature diagnostic closure when caring for patients with substance use disorders, and pursue a comprehensive diagnostic workup for acute liver injury that is not clinically consistent with alcohol-associated hepatitis	
2. Managed alcohol programs (MAPs) are a harm reduction practice with promise to reduce health and social harms associated with severe alcohol use disorder, and to help stabilize alcohol use	
3. In the setting of patient-centered care, shared decision-making, and longitudinal relationships, MAPs can be a clinically appropriate and ethical option for patients with alcohol use disorder and liver disease	

In this case, stopping alcohol exposure initially in hospital was reasonable, when Mr. S presented with an undifferentiated acute liver injury and delirium prevented him from participating in discussions about his alcohol use goals. The admitting medical team may have experienced premature diagnostic closure in attributing the liver injury to alcohol-associated hepatitis, and delaying a more comprehensive workup. The diagnosis of a drug-induced liver injury (rather than alcohol-associated hepatitis) and the improvement in Mr. S’ liver enzymes and bilirubin levels in hospital helped support the decision to reintroduce MAP.

### 4. What ethical considerations are relevant to treatment planning in this case?

There are several ethical considerations for the patient and health care team in this case to weigh when considering whether to restart MAP, informed by four principles of medical ethics: beneficence, nonmaleficence, autonomy, and justice. The balance of these principles may change over time, according to Mr. S’ health status, goals, and apparent benefits from MAP. Overall, patient-centered care, shared decision-making, and longitudinal relationships are essential.

Regarding beneficence, MAP benefitted Mr. S, both before and after his hospital admission. Mr. S and his partner reported improved quality of life since starting MAP, including Mr. S maintaining his housing and having no further episodes of alcohol-related injuries, arrest, or non-beverage alcohol consumption. These benefits should be weighed with the principle of nonmaleficence (to do no harm) [40]. Alcohol is toxic to the liver, and in this young patient with signs of chronic liver disease continued heavy alcohol use may soon result in cirrhosis. However, binge drinking and non-beverage alcohol use are likely worse for liver health. Mr. S’ stated intention to continue drinking, his illness course, and the study by Stockwell and colleagues [16], all suggest that he and his liver were more likely to do better in MAP than out of MAP, at least in the short-term. Longitudinal relationships and assessments are important, as the balance of potential benefits and harms and Mr. S’ goals change over time. Mr. S would be followed closely by the MAP team, and any changes in his health status (including the development of cirrhosis) would be an opportunity to revisit considerations around decreasing or discontinuing his alcohol dose.

Mr. S had capacity to make medical decisions and chose to continue MAP, consistent with the ethical principle of autonomy. He understood and appreciated the potential consequences of both restarting MAP and of stopping MAP. Beliefs about relationships between addiction and autonomy, capacity, or free-will are complex and contested, especially under the biomedical framing of the “brain disease model of addiction” [41–43]. MAPs are informed by a harm reduction philosophy, which promotes autonomy, choice, and compassion [4, 14, 44–49]. Regarding the ethical principle of justice, MAP was compatible with Mr. S’ rights and the law. Enrolling in MAP facilitated access to other health and social benefits (including housing) that he was less able to access without MAP, which is consistent with social justice [11, 14, 48, 50]. Mr. S’ MAP was supported by public funding and MAP has been shown to be cost neutral or cost savings. As a targeted intervention that supports people who are marginalized with multiple care needs, within a spectrum of health and social services for people with substance use disorders, this is consistent with health equity and the public health framework of proportionate universalism [51, 52].

## Conclusion and lessons learned

This case of a 37-year-old man hospitalized with acute liver injury, while enrolled in a managed alcohol program for severe alcohol use disorder, demonstrated several lessons (Table 3). Clinicians should be mindful of premature diagnostic closure when caring for patients with substance use disorders, and pursue a comprehensive diagnostic workup for acute liver injury that is not clinically consistent with alcohol-associated hepatitis. In this case, a rare and addressable cause of acute liver injury was identified and resolved. Managed alcohol programs (MAPs) are a harm reduction practice with promise to reduce health and social harms associated with severe alcohol use disorder, and to help stabilize alcohol use. More program development and research are needed to refine eligibility criteria and programmatic features to make them most effective, and some of this work is already underway (e.g. the Canadian Managed Alcohol Program Study [20]). In the setting of patient-centered care, shared decision-making, and longitudinal relationships, MAPs can be a clinically appropriate and ethical option for patients with alcohol use disorder and liver disease.

## Data Availability

Not applicable.
